# Essential Oils as Alternative Green Broad-Spectrum Biocides

**DOI:** 10.3390/plants13233442

**Published:** 2024-12-08

**Authors:** Fulga Tanasă, Marioara Nechifor, Carmen-Alice Teacă

**Affiliations:** 1 Polyaddition and Photochemistry Department, “Petru Poni” Institute of Macromolecular Chemistry, 41A Gr. Ghica-Voda Alley, 700487 Iasi, Romania; ftanasa@icmpp.ro (F.T.); nechifor@icmpp.ro (M.N.); 2 Center of Advanced Research in Bionanoconjugates and Biopolymers, “Petru Poni” Institute of Macromolecular Chemistry, 41A Gr. Ghica-Voda Alley, 700487 Iasi, Romania

**Keywords:** essential oils, biofilm, healthcare applications, pest control, food packaging systems, cultural heritage protection

## Abstract

Natural compounds from plants represent suitable options to replace synthetic biocides when employed against microorganisms in various applications. Essential oils (EOs) have attracted increased interest due to their biocompatible and rather innocuous nature, and complex biological activity (fungicide, biocide and anti-inflammatory, antioxidant, immunomodulatory action, etc.). EOs are complex mixtures of derived metabolites with high volatility obtained from various vegetal parts and employed to a great extent in different healthcare (natural cures, nutrition, phyto- and aromatherapy, spices) and cosmetics applications (perfumery, personal and beauty care), as well as in cleaning products, agriculture and pest control, food conservation and active packaging, or even for restauration and preservation of cultural artifacts. EOs can act in synergy with other compounds, organic and synthetic as well, when employed in different complex formulations. This review will illustrate the employment of EOs in different applications based on some of the most recent reports in a systematic and comprehensive, though not exhaustive, manner. Some critical assessments will also be included, as well as some perspectives in this regard.

## 1. Introduction

Essential oils (EOs) represent concentrated hydrophobic mixtures of natural organic compounds comprising mainly terpenes, terpenoids, and phenylpropanoids isolated from many vegetal parts of plants (e.g., flowers—rose, chamomile, jasmine, lavender; leaves—peppermint, rosemary, thyme, patchouli; fruits—lemon; roots—vetiver, elecampane; seeds—fennel, anise, cumin, nutmeg; grasses—lemongrass; rhizomes—ginger; bulbs—garlic;) and wood species (e.g., wood as whole—cedarwood, sandalwood; barks—cinnamon, sassafras; leaves—eucalyptus; peels—lemon, lime, orange, tangerine, grapefruit, bergamot; natural resins—frankincense, myrrh; berries—juniper; tree blossoms—ylang-ylang; flowers—clove, orange; heartwood—agarwood) [1].

**Figure 1:**
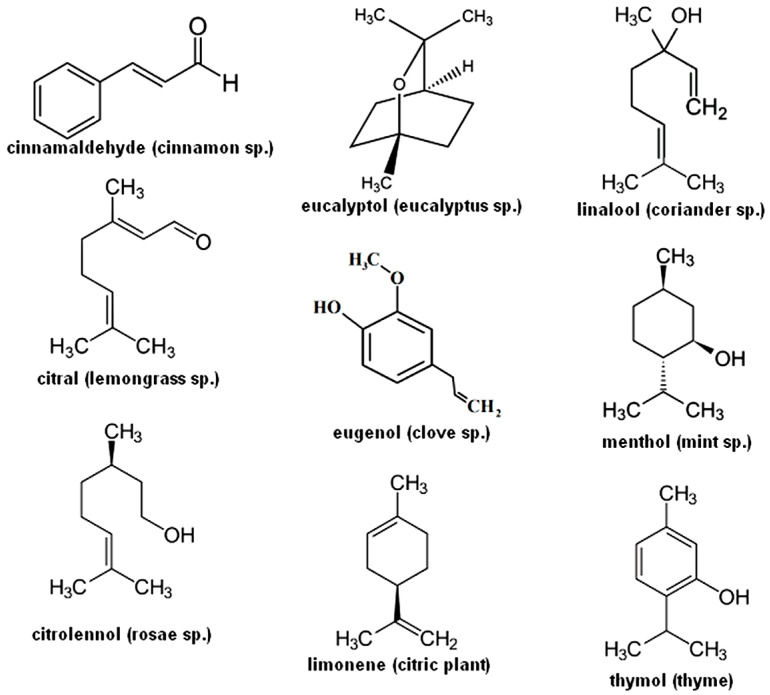
Chemical structures of main components in EOs from different plants.

It is well known that plant extracts usually consist of dozens of organic compounds and only one of them actually has biocide activity [2]. For example, cinnamaldehyde is the biologic active compound in cinnamon oil, thymol in thyme oil, linalool in coriander oil, citral in lemongrass extract, etc. The structures of the main biologic active compound in EOs extracted from different plants are presented in Figure 1.

Some species have high versatility and, therefore, we find them used in a wide range of applications. For example, elecampane (*Inula helenium*), a native species from Eastern Europe, is mostly used in the treatment of respiratory and urinary infections, some digestive syndromes, and also for skin diseases [3]. At the same time, it has been shown that chemical components from *Inula helenium* root essential oil, namely sesquiterpene lactones, alantolactone, and isoalantolactone, possess a very complex biological activity that includes antiproliferative, anti-inflammatory, and antioxidant properties, as well as the capacity to activate enzyme-assisted detoxification, as previously reported [3,4,5,6,7]. 

**Scheme 1:**
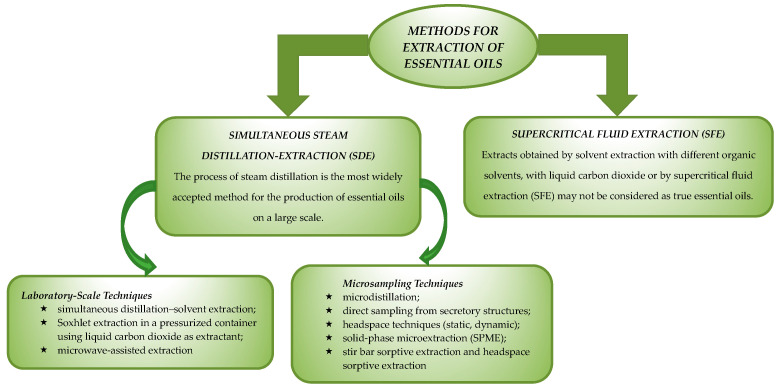
Methods for extraction of EOs (adapted after [8]).

Due to their complex chemical composition and consequently diverse properties, different distillation methods can be employed for the extraction of essential oils from plants, as illustrated in Scheme 1.

The most applied technique is hydro-distillation, a traditional method for extraction, in which the appropriate vegetal material is usually boiled in water [9]. Thus, high-boiling-point organic compounds are effectively separated from their respective mixtures. The oil is vaporized at the boiling point of water or lower, and the resulting hot vapors are further cooled down and condensed until the formation of a biphasic liquid. This method of extraction is preferred considering aspects such as economic (low-cost technique, simple instrumentation) and environmental benefits (it is free from organic solvents) as well.

One of the main applications of essential oils is their use as natural, environmentally friendly alternative biocides. A biocide exerts its effects directly on the target microorganisms, as well as indirectly by preventing the formation and/or destroying the biofilm—the sole mediator of all interactions between support and the surrounding microbiota. The Eos’ biocide activity is a sum of complex phenomena, among which preventing the formation of the biofilm (or even the biofilm degradation) in an effective manner has a primary role in regard to fighting against microorganisms. Thus, EOs have been used as biocides to protect the food surface [10], or for medical and health purposes (aromatherapy and natural cures, cleaning products, cosmetics, and personal care) [11,12], as well as in agriculture as herbicides and for pest control [13], and even for the protection and conservation of cultural heritage [14]. In the first mentioned case, the EOs are included as additives in biodegradable films and coatings for active food packaging [15], which can provide a long shelf life and preserve the good quality of food products during storage by preventing inherent degradation phenomena which may occur under different detrimental circumstances (e.g., proliferation of microorganisms on biofilm, exposure to chemical contaminants, moisture or light conditions). Eos’ specific action in such applications is related to their significant antimicrobial activity (conferred mainly by terpenoids and phenol compounds from their composition) and is manifested as a complex phenomenon, including molecular mechanisms and intermolecular interactions which have been comprehensively reviewed and discussed [16]. The beneficial effects of EOs are also related to their pro-oxidant effects at the cellular level [17].

EOs represent an effective biological strategy applied in order to prevent biofilm occurrence, and their action is manifested through the regulation of proteins coded directly by genes implicated in motility, quorum sensing (QS, known as the cell-to-cell signaling process) [18], and exo-polymer substance (EPS) matrices [10]. Nevertheless, chemical strategies using different biocides (oxidizing, non-oxidizing) and disinfectants are efficient ways to remove biofilms [19]. Such chemical diversity is strongly related to the multifarious interactions, which may add to the mechanical stability of the biofilm, making it possible to employ complex approaches (chemical and mechanical treatments acting in synergy) in order to eliminate the biofilm [20].

EOs’ ability to impair the formation of a biofilm has attracted more and more interest considering that they can effectively contribute to reducing the dose of antibiotics [21].

**Figure 2:**
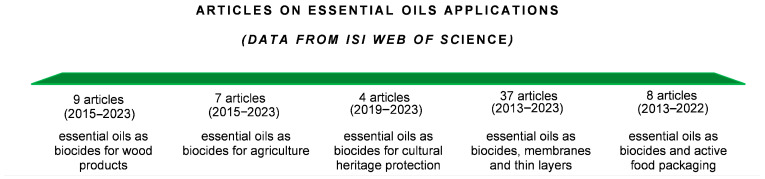
A survey of Web of Science Core Collection related to the articles published in the period 2013–2023 on the addressed topic.

The worldwide interest in replacing synthetic biocides with highly specialized natural ones in various applications, from pest control in agriculture to antimicrobial food packaging, and from healthcare to protection of the cultural heritage, has significantly increased. A solid indication of this interest is the number of articles published in the last decade (although only 16 review articles), as summarized in Figure 2.

Therefore, this review will illustrate the general effort to limit or even to eliminate synthetic biocides from different applications based on some of the most recent reports. It will also provide an appraisal and up-to-date background of the topic and a comprehensive, though not exhaustive, range of applications where EOs have been successfully replacing synthetic biocides. Some critical assessments will also be included, mainly concerning new and complex approaches in the field, as well as some of the EO limitations (e.g., their photochemical and oxidative stability, selectivity, etc.). At the same time, some perspectives in this regard will be presented in order to optimize future research strategies.

## 2. Biofilm-Mediated Specific Interactions Between Microbiota and EOs Biocides

Although the microbiota is a widely unexplored ecosystem, it is of particular importance in human health and diseases, and the modulation of this complex is of the utmost consideration and a biomedical priority. In recent years, the huge problem of antibiotic resistance has been tackled with numerous antibacterial alternatives that have also responded to people’s demand for natural therapeutic products that have no side effects, such as dysbiosis and cyto/hepatotoxicity. Energy metabolism, infection, nutritional status, immunity, behavior, and response to stress form the normal physiology of the host, which is controlled by the microbiota [22]. Homeostasis at the level of the intestinal mucosa and beyond is provided by a well-balanced intestinal microbiota [23].

The mechanisms responsible for the beneficial or harmful influences of the microbiota on the host are still largely undefined, even though its composition and physiology have been intensively researched. Investigation of high-throughput human multi-omic data, such as metagenomic and metabolomic data, together with measures of host physiology and mechanistic experiments in humans, animals, and cells evidences the intricate signaling pathways between the host and different microbiota species [24].

Usually, a higher diversity of the microbiota correlates with a state of health, while a lower bacterial diversity has been observed in people with metabolic disorders, type 1 and 2 diabetes, psoriatic arthritis, celiac disease, atopic eczema, arterial stiffness, and obesity. The microbiota is in a continuous dynamic and it undergoes alterations many times in a human life. It varies greatly from one individual to another, is susceptible to exogenous and endogenous changes, and has an additional impact on the health of the host [25]. The microbiota is influenced by several molecular factors: (1) host-derived factors, which include specific microribonucleic acids, and nonspecific molecules, such as antimicrobial peptides, mucus, immunoglobulin A, and hormones [26]; (2) microbial factors, such as lipopolysaccharides, metabolites, short chain fatty acids, and quorum sensing signaling molecules [27]; (3) dietary molecules (i.e., fats, sugars, proteins); (4) medications (mainly antibiotics, but any drugs could impact the gut microbiota, including natural products utilized in traditional medicine, prophylaxis, or immune stimulation); and (5) various nano- and microstructure particles, which could reach the gastrointestinal tract accidentally by ingestion or by means of oral therapy [28]. The diet is the most important modeling agent for gut microbiota [29].

Different edible (or not edible) substances such as essential oils (EOs) and plant extracts which could be ingested in various situations induce changes into the composition of the gut microbiota, and its modulation [30,31] (Li et al. 2018; Unusan 2020). At the same time, the gut microbiota is impacted by various micro- and nanoparticles currently present in the environment, dust, air, and water, as well as medication and self-care products (mouthwashes, tooth cream, sunscreen, etc.) (Xie et al. 2022).

Complementary and alternative medicine engages drugs derived from plants for therapeutic purposes, used by approximately 75% of the world’s population, especially against metabolic diseases in which the microbiota plays an important role, such as obesity [32].

Essential oils obtained from fruits, aromatic plants, and spices are used to improve human health due to their multiple biological activities [31]. For example, gastrointestinal infection, inflammation, metabolic diseases, and carcinogenesis are the main targets of fruit EO therapy for healthy intestinal functions in humans [29]. EOs are usually colorless liquids at room temperature obtained by plant extraction with water or organic solvent and they are highly volatile plant metabolites [1,31]. EOs are complex mixtures of up to 200 different organic compounds in low amounts (<1%), such as hydrocarbons and their oxygenated derivatives. The interaction between fruit EOs and the gastrointestinal microbiota (GM) involves the modification of the intestinal environment through the microbial metabolism of the bioactive compounds in the EOs or through the compounds excreted by the host. These changes influence the composition and density of the microbiota, but interferences with inevitable impact on the health of the host can also be generated, such as altering the ability of the microbiota to synthesize certain metabolites important for the host (i.e., SCFA and vitamins) [33,34]. 

Complex omics platforms have been used to investigate the effects of EO bioactives on host microbiota and metabolism, leading to the recent development of metabolomic analysis. The specific interactions between fruit EOs and the host microbiota are currently studied in stool microbiota with free bacteria or blood samples, and not on mucosal biopsies with adherent bacteria that cover the entire microbiota which may make results inaccurate. Different regions of the gut (i.e., adhered to gastrointestinal mucosal proteins and epithelial cells) and outside the gut (i.e., central nervous system, systemic immune system, blood metabolites, etc.) are currently being investigated in various animal models to monitor microbiota–EO interferences [35].

Several plants and fruit peels such as pine, citrus, thyme, cannabis, hops, etc., contain terpenes, aromatic compounds with pharmacological and therapeutic properties, namely anti-inflammatory, antioxidant, antibacterial, and gastroprotective activity [36]. The citrus family of fruits, such as sweet oranges, grapefruits and lemons, contains d-Limonene, an active component that improves metabolic parameters and modulates the intestinal microbiota [37].

Polyphenols are a large class of bioactive phytochemicals present in most fruits and are consumed as antioxidants [38]. They are secondary metabolites with over 10,000 structural variants, and they include flavonoids (flavonols, flavanones, flavanols, flavones, isoflavones, and anthocyanidins) and nonflavonoids (phenolic acids, stilbenes, coumarins, xanthones, lignans, and curcuminoids). Some of them (flavanones, flavonols, isoflavone, anthocyanins, proanthocyanidins, and resveratrol) are studied for their significant influence on GI tract function, impacting on insulin signaling, downregulation of oxidative stress, gut bacteria modulation, improvement of endothelial dysfunction, modulation of intestinal absorption, and metabolism [39].

Generally, inter-individual variation in the microbiota pattern and metabolism is important for the health benefits of phytochemicals because the bioactivity of polyphenols can be influenced by their microbial metabolism [40]. Phytoestrogens (stilbenes, coumestans, isoflavones, ellagitannins, and lignans) are polyphenols found in numerous plants, such as soy beans, flaxseed and other seeds, cereals, vegetables, fruits, tea, chocolate, etc. These compounds have a chemical structure similar to human estrogens, but they can exert both estrogenic and antiestrogenic effects.

Isoflavones, ellagitanins, and lignans are metabolized by intestinal bacteria to produce equol, urolithins, and enterolignans, having more estrogenic/antiestrogenic and antioxidant activity in comparison with their precursors. In addition, these metabolites have anti-inflammatory, antiproliferative, and apoptosis-inducing effects and exhibit efficiency against certain diseases, such as cancer, cardiovascular disease, and osteoporosis, as well as menopausal symptoms [41,42]. Ursolic acid is a natural pentacyclic triterpenoid, present in fruit peels (apples, prunes, bilberries, cranberries, hawthorn) and in many herbs and spices (rosemary, thyme, basil, oregano, peppermint, lavender), with anticancer, antidiabetic, antiarrhythmic, anti-hyperlipidemic and antihypercholesterolemic, antimicrobial, hepatoprotective, and hepatoregenerative properties [43,44].

Essential oils (EOs) have received increased attention because of their multiple biological attributes like antifungal, antiviral, anti-inflammatory, antimicrobial, antioxidant, wound healing, anti-parasitic, anti-mutagenic, anti-quorum sensing, antibiofilm, and immune-modulatory activities, and their pharmacological effects against resistant pathogens and increased efficiency against multidrug-resistant and biofilm embedded microorganisms [45].

Natural products extracted from plants offer better biocompatibility and fewer side effects on the human body in comparison with synthetic antibiotics. They have been largely employed to treat infections caused by resistant microorganisms and, therefore, they may represent potential substitutes to conventional drugs [46,47,48,49].

EOs are complex mixtures of volatile secondary metabolites obtained from various parts of the plant, such as flowers, fruits, buds, rhizomes, seeds, leaves, stems, twigs, roots, bark, and wood, and they are extensively applied as adjuvants for healthiness purposes [50]. EOs consist of a large diversity of aromatic oily liquids (approximately 3000 EOs are known nowadays) with a versatile composition, including aldehydes, alcohols, ethers or oxides, ketones, esters, amines, amides, phenols, heterocycles, and mainly terpenes obtained by steam or water distillation [51,52]. EOs are also used as raw materials in various fields, such as perfumes, cosmetics, aromatherapy, phototherapy, spices, household cleaning products, beauty and personal care, and nutrition, or natural health treatments. Moreover, EOs may improve the efficiency of some drugs against different microbes [21].

The progress of novel antimicrobial and anti-pathogenic methodologies can be stimulated by the introduction of essential oils as an eco-friendly alternative to antibiotics, acting by mediating the attenuation of bacterial virulence without interfering with microbial development and aiming to regulate bacterial mechanisms in relation to the expression of virulence factors. The strategy applied against bacterial virulence when treating infections is based on generating bacterial pathogens that are more reactive to the host immune system and stopping the infection process caused by bacteria. Prevention is expected with the prevalence of multidrug-resistant bacterial pathogens with no occurrence of novel microbes with high resistance. Used in sub-minimum inhibitory concentrations (MICs), agents acting against virulence can interrupt the bacterial infection without inhibiting bacterial vitality or even killing the bacteria. The production of antibiotics, virulence factors of pathogens, exo-enzymes, and resistance are regulated by the QS system. Therefore, QS (the bacterial communication system) seems to be a promising target for new anti-virulence agents [53,54,55].

Due to their complex composition, multiple mechanisms that probably act synergistically are involved in EOs’ biological effects [10]. Currently, about 300 of the 3000 known EOs are used commercially, and the most commonly used include lemon, peppermint, citronella, eucalyptus, mint, and orange oil. Most essential oils modify cytoplasmic membranes, leading to the release of lipo-polysaccharides due to their hydrophobic constituents. Essential oils affect the fluidity, permeability, and function of the proteins involved in membrane transport, and the composition of fatty acids in the cytoplasmic membrane as well. Processes such as the formation and dispersal of biofilms can be genetically regulated when using specific essential oils. Several traits, such as cell morphology, the structure and composition of the cell wall, cell division, cellular respiration, ion transport, and the energy balance of the bacterial cell, are influenced by different essential oils. In relation to the concerning medical aspects, EOs aim to accomplish two therapeutic targets: (1) prevention of biofilm formation and production of virulence factor and (2) eradication of already established biofilms [56].

### 2.1. Applications of Essential Oils Biocides as Antibiofilm Agents

Literature reports have claimed that 60% up to 85% of all microbial contaminations are associated with biofilms grown on natural, intact, or damaged tissues (skin, mucosa, endothelial cells, teeth surface) or artificial devices (catheters—central venous, peritoneal, or urinary; dentistry materials; heart valves; intrauterine contraceptive devices; contact lenses; other implants) [57]. In their natural environment, the biofilm structure is favorable to bacteria because it offers safety against harmful conditions, enhances the competition for accessible nutritive substances in a specific zone, boosts the acquirement of novel phenotypic characteristics by gene transfer, and increases interactions in the metabolism of different microbial agents [58].

Biofilm formation is a complex process usually developed in four steps: reversible attachment, followed by irreversible attachment, cell division, and EPS excretion. As the biofilm matures, new bacterial species may be introduced before critical mass is reached and cells are released. Bacteria induce biofilm formation and communicate via quorum sensing [59]. Quorum sensing (QS) permits bacteria to synchronize and control gene expression through the formation and detection of extracellular chemicals called auto-inducers [60]. The cumulative regulation of genetic elements as numbers generates several phenotypic changes which aid the bacteria to develop certain activities to adjust to changes in the environment [61].

Certain bacteria and many strains have developed resistance to nearly all currently used antibiotics and chemotherapy agents, termed multi-drug resistance (MDR) [62]. MDR can be defined as non-susceptibility to at least one agent in three or more antimicrobial categories [63]. MDR bacteria and biofilms are ubiquitous phenomena and represent one of the significant hazards to worldwide healthiness with several deleterious medical and economic consequences [64]. The antibiotic resistance has emerged and developed due to the excessive use and misuse of antibiotics, unsuitable and incorrect prescribing, and large-scale utilization in agricultural applications [65].

Biofilms can have both desirable and damaging effects. While the industry has experienced both the positive and negative aspects of biofilm development, clinically, the devastating consequences of biofilms have been observed. During food processing, product contamination can take place as a result of biofilm appearance by spoilage and the occurrence of bacterial pathogens on food contact surfaces, which lowers products’ shelf-life and causes human diseases [66]. A large number of human infections like cystic fibrosis and otitis media are caused by pathogenic bacteria in medical institutions, particularly on artificial devices like catheters [57].

Sessile cells within biofilms present increased protection in the environment because the barrier properties of the sludge matrix hinder the entry of different agents resistant against microbes. The stationary phase dormant zones in biofilms protect bacterial cells, but many antibiotics can penetrate the EPS since antibiotics mainly act through the disruption of microbial processes [67,68,69]. The increased antibiotic resistance of sessile cells compared with planktonic cells generates clinical issues. Sessile cells within a biofilm can be 10–10,000 times more resistant to antibacterial agents than their free-floating counterparts [70].

An important clinical aspect is the fact that bacterial biofilms cause chronic infections due to their increased tolerance to antibiotics and disinfectant chemicals as well as increased resistance to the host immune system, including phagocytic white blood cells and other components of the body’s defense system [71,72]. Many biofilms are present in a variety of microbial infections, including dental infections [73], periodontitis [74], lung infections resulting from cystic fibrosis and facial filling [75], chronic wounds [76], ear inflammation [77], implant-associated infections [78], chronic rhino-sinusitis [79], contamination in intensive care units [80], contact lens infections [81], and human gastrointestinal tract infections [82].

The absence of novel efficient agents against microbes causes the occurrence of multidrug-resistant microorganisms to be regarded as a global challenge. Alternative treatments of infections caused by microorganisms resistant to traditional therapies are highly necessary. Nowadays, natural antimicrobials have attracted scientific interest due to their function in several microorganism control issues [83]. Natural products from plants can be synthesized as a part of their defense system, and these compounds could provide a valuable source of new drug molecules [84].

Different essential oils were investigated in vitro for their anti-QS activities in *Chromobacterium violaceum* at sub-MICs [85]. Peppermint, lavender, cinnamon, and clove oils exhibited an appreciable inhibition of violacein production. At sub-MICs, clove oil inhibited violacein production in the treated bacteria with no relevant vitality loss of bacterial cells up to 0.12%. The reduction in the formation of violacein in bacteria is due to the anti-QS activity of clove oil and not to its antimicrobial properties. At concentrations higher than 0.16%, the reduction in violacein formation is due to the anti-QS activity of clove oil and its antibacterial properties as well. The anti-QS activity was not exhibited by the major constituent of clove oil, namely eugenol.

Clove oil [86], peppermint oil, and menthol [87] in sub-MICs were tested in two in vitro studies to decrease biofilm formation in *Pseudomonas aeruginosa*. The reduction in various virulence factors (elastase, proteases, pyocyanin, chitinase) in sub-MICs proved that the inhibition of biofilm formation is a QS-regulated process. The production of virulence factors, exopolysaccharide production, and swarming motility were highly inhibited by all compounds under study [86,87]. The native AHL (*N*-acyl homoserine lactone autoinducer) production was significantly reduced by up to 56% in *Pseudomonas aeruginosa* cells by clove oil (at 1.6%) [86].

The biofilms of two species of *Vibrio*, i.e., *V. parahaemolyticus* ATCC 17802 and *V. alginolyticus* ATCC 33787, were eradicated by 56% and 55%, respectively, using *Petroselinum crispum* EO at a concentration of 40 mg/mL. *Ocimum basilicum* EO was proved to inhibit biofilms of *V. parahaemolyticus* to 55%, and *V. vulnificus* and *V. cholerae* non O1 to 87.45%, at a concentration of 50 mg/mL [88]. Another study [89] investigated the effect of *Mentha spicata* EO on two *Vibrio* spp., and it was found that the percentage of inhibited biofilm production was 11.6% for *V. alginolyticus* and 40% for *V. vulnificus* using a concentration of 92 μg/mL.

Bay oil, clove oil, pimento berry oil, and eugenol were tested for their antibiofilm activity [90]. All three EOs and eugenol inhibited biofilm formation in concentrations of 0.005% to 99.7% compared to the control. The vitality of the bacteria was reduced by 20% at a concentration higher than 0.01%.

Different EOs were screened for their antibiofilm activity against uropathogenic *Escherichia coli* in an in vitro study [91]. Carvacrol and thymol per se, as well as essential oils containing both of them in different proportions, namely oregano oil and thyme oil, strongly inhibited the biofilm formation of *Escherichia coli* in sub-MICs (at 0.01%). The reductions in biofilm mass were 88.9%, 86.1%, 94.5%, 94.5% for oregano oil, thyme oil, carvacrol, and thymol, respectively, over the control.

The evaluation of differential gene expression of biofilm-borne bacterial cells in *Staphylococcus aureus* ATCC 29213 at a tea tree oil concentration of 1 mg/mL and an incubation time of 60 min was investigated [92]. An in vitro study [93] investigated the biofilm inhibition activity of cinnamaldehyde (CA) and two different cinnamon oils of *Cinnamomum zeylanicum* (EOCz) and *Cinnamomum cassia* (EOCc). In sub-MICs, EOCc inhibited the biofilm formation of *Escherichia coli* (sub-MIC: 0.12 mg/mL) and *Pseudomonas aeruginosa* (sub-MIC: 0.06 mg/mL) to 100%, without disturbing bacterial cell growth. The biofilm inhibitory effect of cinnamaldehyde was mainly attributed to its cytotoxic activity, and not to its anti-QS effect, as confirmed by cell viability studies.

The EO from *Melaleuca bracteata* (MBEO) leaves was investigated for its anti-QS, antibiofilm, and anti-virulence factor activity in *Chromobacterium violaceum* [94]. MBEO (MIC: 10‰); used at sub-MICs of 0.625, 1.25, 2.5, and 5.0‰, it inhibited the production of biofilm mass, violacein, and different virulence factors, respectively. The viability of bacterial cells was not affected. The swarming motility of *Chromobacterium violaceum* cells was meaningfully disturbed at 5. Concentrations of 5.0, 2.5, and 0.625 of MBEO led to a significant decrease in the concentration of the auto-inducer QS signal in *Chromobacterium violaceum* cells, comparative to the control.

Five EOs and four major active compounds were tested concerning their antibiofilm activities in antibiotic-resistant *Staphylococcus aureus* strains [95]. Eugenol and thyme oil revealed a concentration-dependent biofilm inhibition. At concentrations of 0.2% and 12.8% eugenol, the reduction in biofilm formation was found to be 19.4% and 91.6%, respectively. Thyme oil exhibited a reduced antibiofilm activity, comparative to eugenol. The number of bacterial colonies was significantly reduced by thyme oil and eugenol in the highest concentration tested (12.8%). At the same time, the biofilm matrix was destroyed. Microscopy analysis revealed that the effect of both antibiofilm agents is due to their biocidal activity.

Cassia oil and the polypeptide antibiotic colistin were tested for their ability to kill *Pseudomonas aeruginosa* in planktonic cells and in biofilms [96]. Most of the *Pseudomonas aeruginosa* in liquid culture and biofilm was eradicated by cassia oil in concentrations of 0.2–0.4%, while a concentration higher than 100 μg/mL of colistin killed the bacteria inside the biofilm.

The eradication of *Staphylococcus aureus* and *Pseudomonas aeruginosa* in biofilms was demonstrated by oregano oil after 1 h of incubation at concentrations of 0.4 and 1.0 mg/mL, respectively [97]. Oregano oil effectively reduced the bacterial burden by 25–49-fold in comparison to the control, after bacterial inoculation formed early-stage biofilms.

The activity of tea tree EO, *Lavandula angustifolia* (lavender essential oil) (LEO), *Melissa officinalis* (*Melissa* essential oil or lemon balm-MEO), and linalool, linalyl acetate, α-terpineol, terpinen-4-ol on biofilms formed by *S. aureus* and *E. coli* reference strains demonstrated that MEO, α-terpineol, and terpinen-4-ol showed a higher antibiofilm effect than LEO and its major components, i.e., linalool and linalyl acetate [98]. The tests proved that the *E. coli* biofilm was more susceptible than the *S. aureus* biofilms to the action of EOs, especially to TTO, which destroyed it after 1 h exposure to a 0.78% concentration, contrary to the opinion stating that Gram-negative microorganisms are more resistant to EOs. In comparison with LEO and TTO, the MEO effect is more dependent on the action time. The in vivo tests on biomedical surfaces of urinary catheters and tracheal tubes showed that TTO and terpinen-4-ol used at 2xMIC caused visible biofilm eradication, while increased concentrations were required to eradicate the microbial biofilm on surgical mesh [98]. Another study on the antimicrobial action of TTO against *S. aureus* clinical strains in different growth phases, including stationary phase and biofilms, found that the minimum biofilm eradication concentration was usually 2x CMI, lower than 1% *v*/*v*. The inhibition of biofilm took place in 15 min at a TTO concentration >1% *v*/*v* [99].

The antifungal efficiency of the EO of *Mentha piperita* against *C. albicans* and *C. dubliniensis* biofilm was tested in a study which revealed that biofilm formation was inhibited at a maximum concentration of 2 μL/mL in a dose-dependent manner. This effect is due to the increased concentration of this EO in menthol, which can be incorporated into the fungal cell membrane; the phenolic monoterpene, bearing a hydroxyl group on the phenolic ring, also exhibits an antimicrobial effect due to the cytoplasmic membrane disruption [100].

Eighty-three EOs were screened for their antibiofilm activity [101], and some EOs, namely bay (*Pimenta racemosa*), cade (*Juniperus oxycedrus*), cedarwood (*Calocedrus decurrens*), frankincense (*Boswellia carterii*), lovage root (*Levisticum officinale*), oregano (*Origanum vulgare*), sandalwood (*Santalum album*), thyme red (*Thymus vulgaris*), and Vetiver Haiti (*Cymbopogon martini*) inhibited the *S. aureus* biofilm at a 0.01% (*v*/*v*) concentration. Others, like black pepper (*Piper nigrum*), cananga (*Cananga odorata*), and myrrh (*Commiphora myrrha*) oils exhibited a strong antibiofilm activity at a sub-MIC. One active compound was *cis*-nerolidol (0.01% (*v*/*v*)), which proved to be more efficient than *trans*-nerolidol contained in the three EOs, which inhibited more than 80% versus 45% of the *S. aureus* biofilm growth.

The antibiofilm activity of essential oil from *Satureja hortensis* was tested on *Candida*, *Staphylococcus,* and periodontal bacteria biofilms. The antimicrobial effect of carvacrol inhibited *Candida* and *Staphylococcus* biofilms at 0.03% and 0.06% concentrations. The growth inhibitory effect against periodontal bacteria and the antibiofilm effect in sub-MIC concentration were registered [102]. *S. epidermidis* is one of the main nosocomial agents of indwelling medical devices’ biofilm associated infections (BAIs). Farnesol produced a significant destruction of *S. epidermidis* biofilm structure and an important reduction in biofilm thickness [103]. The EOs from *Ferula asafetida* and *Dorema aucheri* (belonging to *Apiaceae* family) were tested for their activity against *P. aeruginosa* biofilm formation using 25 µg/mL concentrations. *Ferula* EO decreased pigmento-genesis, protease, and biofilm development, while *Dorema* EO affected only pyoverdine and elastase production [104]. The EOs of *Cinnamomum burmannii* and *Massoia aromatic* are another source of antibiofilm agents, proving to inhibit both *P. aeruginosa* and *S. aureus* biofilms. The efficiency of these EOs is based on the presence of cinnamic aldehyde and massoia lactone, respectively, in their structure [105]. Biofilms formed by S. aureus and P. aeruginosa respiratory isolates and reference strains were inhibited in both initial phases as well as maturation by the EOs of *Eucalyptus smithii* and *Juniperus communis* [106].

Several EOs from the Lamiaceae and Apiaceae families (*Ammi visnaga*, *Ammoides verticillata*, *Artemisia arborescens*, *Dittrichia graveolens*, *Lavandula dentate*, *Lavandula multifida*, *Mentha piperita*, *Origanum vulgare*, *Rosmarinus eriocalyx*, *Thymbra capitata*), rich in oxygenated monoterpenes (mostly alcohols, such as thymol, carvacrol, and linalool), were investigated for their antibiofilm effect. The EOs from *T. capitata* and *O. glandulosum* (0.75–1.5%) inhibited *E. faecalis* biofilms, similar to those extracted from *A. verticillata* and *L. multifida* (1.50–3.00%). The administration of EOs proved to be more efficient than the administration of the main component itself [107]. The EO from *Baccharis psiadioides* (*Asteraceae* family) is known for its antipyretic and anti-inflammatory properties, and as a snake bite antidote. At the same time, it has been proved to exhibit antimicrobial and antibiofilm action on 13 resistant *E. faecalis* strains [108].

Eugenol, a major compound in clove (*S. aromaticum*) EO, acts by disrupting cellular membrane permeability, and citral, containing geranial (*trans*-citral, citral A) and neral (*cis*-citral, citral B), found in the citrus plants leaves and fruits, affects both the cytoplasmic/outer membrane as well as the stress response mediated by the sigma factor RpoSin *E. coli* [109]. The preformed biofilms of different *Candida* spp., excepting *C. glabrata,* were inhibited by the *Thymbra capitata* EO at 2xMIC, probably due to the high content in phenols (carvacrol) [110]. Ten terpenes, the main components of EOs (carvacrol, citral, eucalyptol, eugenol, farnesol, geraniol, linalool, menthol, γ-terpinene, and thymol), were tested on different *Candida* strains (*C. albicans*, *C. parapsilosis*, *C. glabrata*). The best results were obtained using carvacrol against *C. albicans*, *C. glabrata*, and *C. parapsilosis* biofilms, but good results were recorded for geraniol and thymol [111].

The antibiofilm effect of *Citrus limon* and *Zingiber officinale* EOs were investigated, and it has been shown that they can be used against biofilms of *Klebsiella ornithinolytica*, *K. oxytoca* and *K. terrigena* [112]. Dual-species biofilms produced by *L. monocytogenes* SZMC 21307 and *E. coli* SZMC 0582 were eradicated by their treatment with *Cinnamomum zeylanicum* EO at concentrations of 1 mg/mL [113]. Using *Origanum majorana* EO and *Thymus vulgaris* EO, the inhibitory effect was detected at 0.5 mg/mL and 1 mg/mL concentration, respectively. These values were much lower than those recorded in the eradication of monoculture biofilms. All studied EOs decreased biofilm formation but at concentrations higher than those required for monospecific biofilms eradication.

A comprehensive study on the synergistic effect of EO and antibiotics proved that the association of EOs with antibiotics was beneficial [114]. EOs of *Cinnamomum zeylanicum*, *Mentha piperita*, *Origanum vulgare*, and *Thymus vulgaris* were associated with norfloxacin, oxacillin, and gentamicin, and their combined activity was investigated on bacterial biofilms produced by *S. aureus*, *S. epidermidis* IG4, and *E. faecalis*. The results showed that all EOs had a synergistic effect. The advantages of combined therapy are obvious: the decrease in antibiotic doses and implicitly reducing the resistance to antimicrobial drugs.

Essential oils from 21 plants were investigated for their activity against 20 fluconazole-resistant strains of *Candida albicans* fungus. The oils of *Cymbopogon martini* exhibited strong inhibitory activity with MIC_50_ in the 90–100 μg/mL range due to their main constituents, citral and cinnamaldehyde, respectively. The test oils exhibited remarkably synergy with fluconazole or amphotericin B and were more effective for the inhibition of azole- and amphotericin B-resistant strains than fluconazole and amphotericin B [115].

Antiquorum sensing and antibiofilm activity for seed extract from *Trigonella foenum-graecum* L. (Fenugreek) were reported, and sub-MICs were tested in *Pseudomonas aeruginosa* strains [116]. *T. foenum-graecum* seed extract inhibited the AHL regulated virulence factors to a large extent, including protease, LasB elastase, pyocyanin production, chitinase, EPS, and swarming motility. *T. foenum-graecum* seed extract decreased the biofilm forming ability of the pathogens in tested strains in high levels. Caffeine, the major compound of *T. foenum-graceum* extract, reduced the biofilm formation and occurrence of QS regulated virulence factors at a 200 µg/mL concentration level [116].

### 2.2. Essential Oils Biocides in Dentistry

Dental plaque biofilm plays an essential role in oral pathology, the etiology of dental caries, but also in the contamination of dental materials surfaces, such as those used in implant and prosthetic rehabilitation (implants, impression materials, alloys for prosthetic use, etc.).

Chemical substances as medications with both antibacterial and antibiofilm activities are not biologically friendly to the dental and peri-radicular tissues. In the recent years, research on the use of natural products for root canal disinfection and removing the smear layer and the microbial biofilms formed in the mouth has gained importance [117]. *E. faecalis* is commonly recovered from teeth with persistent endodontic infections, creating biofilms attached to the canal walls or located in isthmuses and ramifications from where are difficult to eliminate by current substances, such as sodium hypochlorite and chlorhexidine [118]. Chloroform solutions of eucalyptus and orange EOs associated with cetrimide at concentrations varying from 0.05% to 0.3% reduced the biofilm by 70–85%. The two EOs enhanced the efficiency of cetrimide, which effectively eradicated the biofilms in lower doses, the synergic effect probably being due to lipophilic compounds (e.g., terpenoids or phenolics). The *Melaleuca alternifolia* EO presents an antibacterial effect and is very effective against oral *S. mutans* biofilm, decreasing the gingival bleeding index. Mouthwashes with this EO decreased the total oral bacteria counts. The EO was used at 5% concentration without side effects [119]. Carvacrol and oregano oil are known for their effect on *Staphylococcus strains.* They showed in vitro effects on staphylococcal biofilms, and the biofilm inhibitory concentrations had 2–4xCMI values. Thymol is used in mouthwash, with anti-plaque effects due to the biofilm matrix destabilizing effect [120].

The use of EOs (menthol, thymol, and eucalyptol) for oral health proved to be beneficial by preventing the biofilm formation in patients with prostheses [121]. In several cases, these EOs were more efficient than cetyl-pyridinium chloride [122]. EOs can be used daily, in the long-term, for reducing the supragingival plaque and gingivitis [123]. EOs prevented plaque-like biofilm development for 7 h after mouthwash, presenting a possible alternative to chlorhexidine for the pre-surgical rinse or after periodontitis treatments [124]. Mouthwash containing EOs rich in eucalyptol, methyl salicylate, menthol and thymol, combined with ethanol at high dilution, exhibited an increased antibiofilm activity. The nonalcoholic mixture of EOs tested on *Aggregatibacter actinomycetemcomitans* strains had better anti-planktonic behavior [125].

A promising approach for the treatment and prevention of caries consisted of combinations of these EOs with xylitol in mouth rinse against *S. mutans*-derived biofilms [126]. EOs from *Mentha piperita* and *Rosmarinus officinalis* proved to be effective against *S. mutans*, one of the main agents of dental caries. *Mentha piperita* EO (having a menthol concentration below 3.6%) was more effective than rosemary oil (containing piperitone as the main component) and chlorhexidine (at 4000 and 8000 ppm). The use of toothpaste blended with EOs indicated that lower concentrations of the EOs were more effective than chlorhexidine [127]. The association of chlorhexidine with EO is indicated for better antibiofilm activity in oral treatment [128]. Other alternatives to chlorhexidine were represented by eugenol and citral at subinhibitory concentrations, when they influenced biofilm formation and the virulence of methicillin-susceptible *S. aureus*, MRSA, and *L. monocytogenes* strains, with a low-risk for selecting resistance [129].

The effects of EOs obtained from twenty medicinal and aromatic species on biofilms produced in vitro by different microbial strains were compared with the results recorded with nystatin and chlorhexidine digluconate. The *Aloysia gratissima* and *Coriandrum* spp. EOs strongly inhibited *C. albicans*, *Fusobacterium nucleatum*, *P. gingivalis*, *S. mitis*, and *S. sanguis*. The *C. articulates* EO inhibited *F. nucleatum* and *P. gingivalis* biofilms. *A. gratissima* inhibited the *S. mitis* biofilm more intensively than chlorhexidine [130]. The antibiofilm efficacy of *Cymbopogon martini* and *Thymus zygis* EOs was tested on the multispecific biofilms of *S. mitis*, *S. sanguinis*, and *E. faecalis* in the root canals of extracted teeth. The addition of an oil-based irrigant to 1.5% sodium hypochlorite proved to be more efficient against biofilm development [131]. A good antibiofilm and anti-caries effect, comparable to that of chlorhexidine (0.12%), was observed for a mouth rinse containing *Matricaria chamomilla* L. EO. The antibiofilm effect has been evaluated as a decrease in Colony-forming units (CFUs) for total *S. mutans*, *S. sobrinus* and *Lactobacillus* sp., and the anti-caries effect has been studied as the effect on enamel demineralization compared to phosphate-buffered saline solution. Mouthwash containing *Matricaria chamomilla* EO produced a mineral loss reduction of 39.4%, which was very close to that of chlorhexidine (47.4%) [132]. The *Citrus limonum* and *C. aurantium* EOs exhibited an antibiofilm effect comparable to 0.2% chlorhexidine but lower than 1% sodium hypochlorite on multi-specific biofilms formed by *C. albicans*, *E. faecalis*, and *E. coli* [133].

The EOs from *A. gratissima*, *Baccharis dracunculifolia*, *C. sativum*, and *Lippiasidoides* demonstrated a potent inhibitory activity *on S. mutans* biofilm, probably due to the presence of compounds such as thymol, carvacrol, and *trans*-nerolidol [134]. Mouthwashes containing *Citrus hystrix* leaf EO alone or in combination with chlorhexidine inhibited the periodontopathogenic bacteria and *S. sanguinis* and *S. mutans* biofilms [135]. The *B. dracunculifolia* EO reduced the growth rate of *S. mutans* biofilm at the same level as triclosan (one week of use) [136]. EO from *Curcuma longa* inhibited the growth, acid production, and *S. mutans* adherence to saliva-coated hydroxyapatite beads and biofilm development [137]. The *Melaleuca alternifolia* (tree), *Eucalyptus radiate* (eucalyptus), *Lavandula officinalis* (lavandula), and *Rosmarinus officinalis* (rosmarinus) EOs significantly inhibited adhesion of *S. mutans* (>50%). Tea tree oil and manuka oil significantly inhibited the adhesion of *P. gingivalis* [138]. EOs from *Coriandrum sativum* exhibited an inhibitory activity against *C. albicans* oral isolates from patients with periodontal disease, similar to nystatin, suggesting its promising potential for the prophylaxis and treatment of oral candidiasis [139]. Due to its major components (decanal and *trans*-2-decenal), *C. sativum* EO could bind membrane ergosterol, similarly to nystatin and amphotericin B. The *C. sativum* EO also inhibited the proteolytic activity of *C. albicans* and affected the normal morphology of yeast cells (at 156.0 to 312.50 mg/mL concentration), probably by affecting membrane permeability due to the presence of mono- and sesquiterpene hydrocarbons [140].

Eugenol (90.2%), eugenol acetate (6.5%), and β-caryophyllene (1.3%), the major components of *Syzygiumaromaticum* EO showed a significant inhibition of *S. aureus* biofilm production at a concentration of 0.106 mg/mL [141]. At the same concentration of *Cinnamomum zeylanicum* EO, the biofilm formation *of S. aureus* was significantly reduced, probably due to its components: cinnamaldehyde (86.5%), benzaldehyde (4.2%), cineole (1.7%), cinnamic acid (1.5%), and eugenol (0.1%). Besides the examples presented above, Table 1 summarizes the most used essential oils and their components with QS and activity against biofilms formation, reduction in virulence factor, and eradication of biofilms tested against numerous other types of bacteria.

## 3. Essential Oils as Green Biocides in Agriculture and for Pest Control

Essential oils as well as their corresponding constituents and derivatives are effectively used as antimicrobial reagents in various applications (cosmetics, pharmaceuticals, healthcare, agriculture, food processing, and conservation etc.), exerting biocide activity against a wide variety of pathogenic vectors [162,163,164,165]. These are effective agents against fungi, viruses, bacteria, and insects [166,167,168] given their mainly significant antioxidant properties and action through variable active mechanisms. In relation to their above-mentioned specific activity, essential oils have attracted increasing interest as innovative, environmentally friendly solutions as alternative feasible ways to better protect and preserve wood-based products used in structural applications. Such an approach is beneficial as it involves compounds derived from renewable resources, with some of essential oils exhibiting multi-task action in relation to both environmental and health safe concerns. The use of essential oils as natural pesticides represents a valid alternative, even considering their high volatility which implies a variation in biologically active compounds with the concentration level in the treated wood [169]. Table 2 exemplifies some corresponding activities as efficient biocides for different essential oils, as presented in the literature data.

Essential oils extracted from woody-type plant species, such as coniferous species belonging to the family *Cupressaceae* (namely, juniper, and cypress), tea tree (*Melaleuca alternifolia*), *Eucalyptus* species from the myrtle family *Myrtaceae*, or softwood species *Cupressus nootkatensis* (yellow cedar) presented efficient fungicide and insecticide activity in experimental trials [170,171,172,173]. Active compounds from essential oils derived from thyme, *Geranium* species, *Lavandula* species (lavender), cinnamon, and oregano present effective biocide action against wood decay caused by different degrading molds [182]. Compounds such as thymol, carvone or carvacrol as aldehyde- and ketone- type forms, or those with phenolic structure, as well as terpenes and their ester forms present in volatile oils, are very efficient in protecting wood surfaces towards basidiomycete-type microorganisms that cause rotting [190,191]. Other compounds presenting essential oils, including geraniol, thymol, carvone, citronellol, and borneol, to a large extent inhibit the germination and development processes for mold spores. 

Antiviral and insect repellent activities were noticed for essential oils extracted from *Meliaceae* or mahogany family [175]. The same effective action as biocide agents was observed for volatile oils derived from well-known healing plants (e.g., cloves—aromatic flower buds of the tree *Syzygium aromaticum*, thyme—*Thymus vulgaris*, mint—*Mentha* species, lemongrass—*Cymbopogon* species, cinnamon—*Cinnamomum cassia*, rosemary—*Salvia rosmarinus*, oregano—*Origanum vulgare*) [176]. Essential oils from cassia and cinnamon species, as well as their correspondent extracts, exhibited specific anti-termite and antifungal activity. The same effect was noticed when using wood tar oil when applied in order to conserve wood characteristics, namely, to protect ships against rotting processes, as water repellent in roofing applications, and as antibacterial agents in healthcare applications [162].

Essential oils extracted from vetiver, a perennial bunchgrass (*Chrysopogon zizanioides*) [177], patchouli—a bushy perennial herb (*Paogostemoncablin*) [178], as well as orange oil—produced by cells within the rind of an orange fruit (*Citrus sinensis* fruit) and extracted as a by-product of orange juice production by centrifugation [179] and cinnamaldehyde compound from cinnamon oil [192] were tested and confirmed to be active agents against termites (*Coptotermes formosanus*), with various results. Essential oils can also be used as fumigants, and the effectiveness of such substances extracted from different vegetal species (e.g., dill weed *Anethum graveolens*, rosemary *Salvia rosmarinus*, lemongrass *Cymbopogon* species, geranium *Geranium* species, tea tree *Melaleuca alternifolia*) is being tested and assessed in experimental trials as repellents against subterranean termites (*Reticulitermes flavipes*—Kollar), wherein insect mortality ranged between 95 and 100% in both formulations [163].

Essential oils can be effectively applied for wood impregnation in order to inhibit mildew activity, as indicated in experiments performed on pine wood specimens that were impregnated with steam-distilled extracts derived from plants including geranium *Geranium* species, lemongrass *Cymbopogon* species, rosemary, ajowan, tea tree *Melaleuca alternifolia*, dill weed, and thyme *Thymus vulgaris*. All these had positive results against *Penicillium chrysogenum*, *Trichoderma viride*, and *Aspergillus niger* microorganisms [180], using both treatment solution (when a protection up to 20 weeks was evidenced using extracts from dill weed, geranium and thyme) and fumigation (when vapors from dill weed were efficient at the same extent as solution). The same plant extracts were applied in tests for the inhibition of decay fungi such as *Trametes versicolor*, *Postia placenta*, and *Gloeophyllum traheum* [181], when the most effective was that derived from thyme.

An important antifungal activity was evidenced for essential oils from clove, sweet flag, and oregano in experiments testing the protection of beech wood samples against molds and decay microorganisms (*Aspergillus niger*, *Penicillium brevicompactum, respectively, Coniophora puteana*, and *Trametes versicolor*), while no efficiency was noticed for oils extracted from savory and birch [165]. 

Another experimental study [182] proved the effectiveness of essential oils extracted from specific plants from Egypt in testing antibiofilm activity against wood rotting microorganisms, namely *Hexagoniaapiaria* and *Ganoderma lucidum*. Plant extracts containing geranium oil, eugenol, and cinnamaldehyde compounds presented significant fungicide activity when tested against biofilm formation caused by fungal microorganisms such as *Trichoderma harzianum*, *Coniophora puteana*, *Ophiostoma floccosum*, *Ophiostoma piceae*, *Oligoporus placenta*, *Androdia xanthan*, *Shaeropsis sapinea*, and *Leptographium procerum* [193].

Essential oils extracted from *Juniperus formosana* (leaf, fruits) were tested as antifungal agents against different wood degrading molds [183], with the most effective being the extract obtained from leaves which contain compounds with significant fungicide action, such as α-cadinol and elemol. The same enhanced effect against fungal biofilm formation was observed when using essential oil extracted from *Cinnamomum osmophloeum* Kaneh, which contains cinnamaldehyde as the most effective fungicide compound [184]. Significant action as antifungal active agent was proved for essential oil compounds such as eugenol and isoeugenol, as well as α-methyl cinnamaldehyde and (*E*)-2-methylcinnamic acid [194]. Considering the chemical structure of compounds that constitute the EOs, it has been proven that reagents which contain functional groups such aldehyde or carboxylic acid, a conjugated double bond, and an alkyl chain pendant to the aromatic ring are involved up to a large extent in the modulation action of EOs as effective fungicide agents. A relevant example is given by the phenylpropene-type compounds, which exhibit enhanced fungicide action (*Laetiporus betulina* and *Laetiporus sulphureus*) mainly due to their structural characteristics, namely methyl functional groups linked to the benzene rings in the *ortho* position.

Overall, essential oils extracted from almost any part of selected vegetal species, trees, or plants have proved to exhibit a variable effective activity against bacteria, fungi, and insects (termites, nematodes). Such effectiveness has been confirmed for EOs derived from flax seeds or linseed *Linum usitatissimum* [185], cinnamon bark *Cinnamomum cassia* [186], citrus peels from orange fruits *Citrus sinensis* [187], tung seeds from tung-oil tree *Vernicia fordii*, and kukui nut (*Aleurites moluccana* or candle nut species) [195], as well as for some by-products, such as lampante oil [189], which results from Istrian white olives during their processing for the production of virgin oil.

**Scheme 2:**
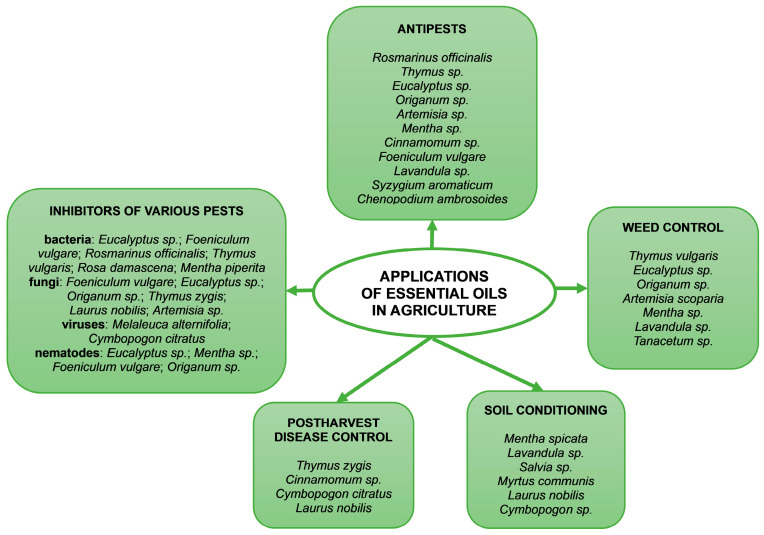
Some examples of plants with biocide activity in agriculture applications through essential oils from their chemical constituents (adapted from [196]).

Essential oils offer also significant potential for agricultural purposes (e.g., protection of stored products, livestock, bees, and crops), since most of them are non-harmful in toxicity to the surrounding environment, including animals [196,197]. Some examples of plants with biocide activity in agriculture applications are schematically presented in Scheme 2.

Despite the considerable advantages of EOs, these also have some disadvantages, such as relatively slow action; reduced effectiveness with time duration; high quantities are required and applied for an increased efficacy (e.g., for weed control that may cause harmful damage to the environment and microbial communities in the soil, they can exert a negative effect on crops, and even partly their destruction). Increased volatility is another significant problem raised when using EOs, and an effective method to control this issue as well as the release properties is EO microencapsulation. This method can provide an effective production of natural biocides which successfully mimic the chemical compartmentalization in plants by protecting essential oils from degradation [198]. The microencapsulation strategy also has beneficial effects in relation to the chemical stability (e.g., oxidative stability, thermostability), shelf-life, and biological activity of EOs [198,199,200,201,202,203,204]. 

## 4. Essential Oils for Antimicrobial Food Packaging Systems

In recent decades, research interest has been remarkably focused on the use of natural products of natural origin (animal—chitosan, propolis; vegetal—essential oils; microbial—lysozyme, nisin, etc.) that have biocide properties in most various applications: cosmetics and pharmaceutics, food preservation and packaging, biomedical engineering, etc. [205,206,207]. Among them, essential oils (EOs) showed high effectiveness against a wide range of bacteria and were significantly safer than their synthetic counterparts, which made them especially suitable for food preservation and packaging, despite their intrinsic disadvantages (complex composition that depends on many parameters—species and cultivar, cultivation area, time of harvesting, storage and transportation; they are highly volatile, liposoluble, and insoluble in water; instable upon exposure to UV-vis radiation when undergoing photochemical and oxidative degradation). Therefore, the pre-processing of EOs is required in order to limit their volatility and instability, but to maintain or increase their biologic activity, ability to interact with food, and dispersibility. Thus, the (micro/nano) encapsulation of EOs [208] or their incorporation in various blends and composite formulations, such as basil oil–chitosan–PVA [209], starch–chitosan–oregano oil [210], or chitosan–gelatin–orange peel oil [211], has been successfully employed.

The antibacterial activity of EOs is of special interest when it comes to their application in food processing and preservation. The most relevant feature in line with this is EOs’ lipophilic, character associated with their hydrophobicity. This allows them to adhere to the phospho-lipidic membrane of bacterial cells and alter its properties as to make it more permeable and, subsequently, cause leakages of ions and other cytoplasmatic elements and metabolites [52]. After a certain loss of volume, bacterial cell death occurs.

Interestingly, EOs can act on a single target or multiple targets. For example, *trans*-cinnamaldehyde has showed an inhibitory effect on the growth of *Escherichia coli* and *Salmonella typhimirium* by accessing the periplasm and penetrating into cells [51]. It was demonstrated that EOs’ antibacterial activity strongly depends on their composition. The highest activity was shown by EOs containing mainly aldehydes and phenols (cinnamaldehyde, citral, carvacrol, eugenol, thymol—it seems that the position of the –OH group on the benzene ring does not affect the biocide activity of phenol-containing EOs), EOs containing terpene alcohols displayed medium activity, while those containing ketones or esters proved to be much less active [212].

Another characteristic of concern is that EOs’ activity against Gram-positive bacteria is higher than for Gram-negative bacteria. This is related to the EOs’ chemical composition and the reduced sensitivity of Gram-negative bacteria biofilm, which explains the high effectiveness of EOs in inhibiting the growth of bacteria such as *Salmonella* spp., *E. coli*, and *Listeria monocytogenes* [52]. At the same time, combining two or more EOs may be beneficial if they act synergistically; otherwise, they can neutralize each other’s effect [213]. They also interact with food preservatives and may intercede in conservation procedures. Some of the following factors have been considered as potential synergists when EOs were employed in food processing and packaging: low pH and oxygen levels (vacuum), medium temperature and high hydrostatic pressure, low water activity, chelators, etc. For example, low oxygen levels (vacuum packaging) have a limiting effect on bacteria metabolism and prevent the chemo-oxidative degradation of EOs (oregano and thyme EOs showed significantly increased activity against *S. typhimurium* and *S. aureus*, while clove and coriander showed completely biocidal activity on *A. hydrophila*) [213].

Some applications of the most well-known EOs are systematically presented in Table 3.

### Modern Approaches

Novel concepts for the use of EOs in food preservation and active packaging comprise the design of edible coatings that are able to preserve the original characteristics of food. These films consist of complex carbohydrates, proteins, and lipids, which provide satisfactory mechanical properties and good barrier characteristics against water and oxygen. It has been shown that the phenolic components of EOs have the highest bactericide activity, as in the case of carvacrol and thymol, which were able to destroy the outer layer of Gram-negative bacteria, or oregano and rosemary EOs, which have showed a strong antioxidative effect on lipids as demonstrated on various types of cheddar cheese (Gram-negative bacteria—*Escherichia coli*, *Salmonella choleraesuis*, *Pseudomonas aeruginosa*, *Yersinia enterocolitica*—and Gram-positive bacteria—*Bacillus cereus*, *Staphylococcus aureus*, *Listeria monocytogenes*, *Enterococcus faecalis*) [52]. Other coating formulations include sodium caseinate, which significantly increased the barrier effect against water and acted as a matrix when cinnamon and ginger EOs were added to the formulation, thus granting their homogeneous dispersion in bulk [231].

Protection against bacteria is a constant concern along the entire chain of food processing. Therefore, understanding biofilm behavior toward various biocides may offer valuable insights into the effective control of biofilm development on food surfaces. Hence, the hurdle technology seems to be a successful alternative as it wisely combines two or more control methods, such as physical–chemical or chemical–biological, aiming at multiple bacteria targets [232].

Thus, it was reported that EOs have increased the efficiency of other disinfectants and sterilization procedures when used in various combinations: peracetic acid and EOs of *Lippia sidoides*, *Thymus vulgaris*, and *Pimenta pseudochariophyllus* [154]; EOs of *Lippia sidoides* combined with *Thymus vulgaris* or peracetic acid [233]; cold nitrogen plasma and clove oil [234] or thyme oil [235].

It is not possible to advance in active food packaging without considering the employment of nanotechnology in the elimination of pathogens along the food processing chain and maintenance of food characteristics during storage [236]. Recent studies have focused on various nano-carriers that are able to incorporate EOs and ensure their homogeneous distribution, at the same time providing good barrier properties, diffusivity, and preservation of food organoleptic features. EOs have been successfully used in such applications. Nanoemulsions were considered for fruit and vegetable coatings: oregano EO was tested on lettuce against *L. monocytogenes*, *E. coli*, and *S. Typhimurium* [237], and carvacrol and eugenol nanoemulsions were tested on spinach against *E. coli* and *S. enterica* [238]. Other films containing EO nanoemulsions have been proven to be effective against *E. coli*: an edible film based on sodium alginate and thyme EO [239], and a carnauba wax film including lemongrass EO [240]. Even EO-based nanoemulsions have been considered for combined techniques in hurdle technology [241].

Nanoliposomes were considered when nanoliposome-encapsulated thymol and carvacrol proved to be effective against *S. aureus* [242,243], and carvacrol encapsulated in nanoliposomes or in polymeric nanocapsules of Eudragit^®^ showed biocide activity against *S. aureus*, *Listeria monocytogenes*, *E. coli,* and *Salmonella* spp. [244].

Polymeric nanoparticles and nanocapsules have been employed as well, but the nanoparticles are preferred when the active compound needs to be released at slow rates as they are adsorbed at the surface of the nanoparticles, not incorporated inside the particle as in the case of nanocapsules [245]. The following various formulations have been successfully tested on the most common bacteria: zein nanoparticles containing thymol [246], carvacrol-loaded chitosan [247], and chitosan-tripolyphosphate nanoparticles [248].

Nanofibers made of natural (proteins, lipids, carbohydrates) or synthetic polymers and containing EOs can be considered for edible coatings and in active packaging. Thus, cinnamon EO has been encapsulated in β-cyclodextrin proteoliposomes, which were subsequently incorporated into nanofibers of poly (ethylene oxide) PEO [249]. This new biocide packaging material proved to be highly effective against *B. cereus* on beef.

## 5. Essential Oils Applications in the Conservation of Cultural Heritage

Biodeterioration describes a complex of phenomena that occur at the interface between the micro-/macrobiota and the corresponding substrate, a system of interrelated reactions and interactions based on the metabolic necessities of the living microorganisms. It can affect different materials (organic—wood, leather, natural textiles, paper and papyrus, natural pigments and dyes; inorganic—stone, marble, metals, glass) found in a wide variety of cultural assets, such as buildings and monuments, books, old documents and historical records, paintings on different substrates, murals and cave imprints, clothing, weaponry, stained glass, etc. [14]. The preservation of the historical and cultural heritage of mankind is of the utmost importance, as it provides opportunities for the next generations to further research, study, and understand the achievements of their ancestors and honor their legacy. Moreover, any kind of artifact should be considered for restoration and conservation, and sustainable, environmentally friendly approaches to prevent, limit, or even combat biodeterioration must be taken into consideration for long-term applications [250,251].

Various methods have been employed to fight against the biodeterioration of cultural heritage, benefiting from the advances in analytical investigations. Thus, mechanical methods have shown limited effectiveness against microbial growth. However, laser techniques have yielded satisfactory results when used for surface cleaning [252]. Other physical treatments have been applied to preserve heritage sites and artifacts, such as the following:

- thermal methods have disinfectant effects [253];
- UV irradiation limited the growth of fungi, algae, and bacteria [254];
- gamma radiation granted site sterilization [255];
- atmospheric plasma torches were effective in cleaning historical stones [256].

Nevertheless, chemical and biochemical methods are the most widely used [257], and the effectiveness of biocides of various classes (quaternary ammonium salts, isothiazoles, phenols, aldehydes, etc.) has been tested [258]. Even the employment of inorganic nanoparticles has been considered, and studies have confirmed their effectiveness in preventing the biodeterioration of cultural heritage [259,260,261,262].

Despite the advances in the aforementioned methods, a new sustainable, environmentally friendly approach has attracted a lot of interest in recent decades, namely the use of natural biocides. Among them, essential oils (EOs) have proven to be effective biocides for the recovery and conservation of heritage objects, monuments, and artifacts, even though their activity has mainly been tested in the laboratory and for short time intervals.

One major concern derives from their complex composition, as they contain numerous organic molecules aside the main active component (see Figure 1). Therefore, the possible long-term interactions of other compounds in extracts with the heritage substrates must be assessed and addressed.

Another issue of interest is the photochemical stability of EOs and their activity after long-term service under photo-oxidative stress [263]. Temperature, UV-vis radiation, and the presence of oxygen are the main factors affecting their stability, since EOs are prone to degradative processes which result in the loss of biologic activity. It has been demonstrated that some EOs, namely eugenol and thymol, have maintained their biocide activity after exposure to UV-vis radiation under controlled conditions, while cinnamaldehyde showed a significant decrease in its inhibitory activity after UV irradiation [2].

Many EOs are active biocides against fungi, bacteria, and insects, and some of them are highly selective. For example, EOs from Thymus and Mentha showed an antifungal activity higher than a commercial fungicide selected as reference [264]; carvacrol specifically inhibited *Staphylococcus aureus* and *Staphylococcus epidermidis* [265]; most Lamiaceae-derived EOs are highly effective fungicides [266]; EOs from *Thymus vulgaris* and *Pelargonium graveolens* have limited the growth of *Aspergillus niger*, *Penicillium chrysogenum*, and *Trichoderma viride*, so that they can be used to prevent the mold colonization of wooden artifacts [181].

By applying the principle of synergy, mixtures of EOs have been designed, with consideration of their chemical composition, and tested. Mixtures of EOs from thyme and oregano are the most effective biocides, given their similarity in composition (they both contain terpenes) [267]. EOs from *Citrus aurantium* L. var. *amara* and *Cinnamomum zeylanicum* (Zege emulsion) were effective against mold colonies in paintings [268]. A complex biofilm made of cyanobacteria, chlorophyte, and green algae (Chlorella) that colonizes stones surface has been treated with EOs from various plants (basil, cloves, eucalyptus, thyme, pine tree, and tea tree), and the results were compared with those of a commercially available biocide, namely Preventol^®^RI50 (ammonium quaternary salts). The EO mixtures showed a higher biocide activity than the individual EOs due to their synergic action [269]. A strong antifungal effect has been confirmed for mixed EOs from oregano, lemongrass, and peppermint (mixing ratio = 1:1:1) when tested on historical papers [270]. However, the interactions of EOs in mixtures have to be further investigated because, at this moment, it is difficult to anticipate their overall biocide activity in order to design a specific formulation for an intended application and expect it to act at satisfactorily.

Some typical applications of EOs in the conservation of cultural heritage are summarized in Table 4.

## 6. Concluding Remarks and Perspectives

The range of various applications where EOs are employed as alternative, green, wide-spectrum biocides (medicine and pharmacy, wood protection, pest control, active food packaging, preservation of cultural heritage) has substantiated the importance of these natural compounds, both theoretically and practically. EOs have the ability to be used in various forms, formulations, and procedures, in indoor and outdoor conditions, and on different (natural and synthetic, organic and inorganic, raw or treated, contemporary or old/ancient) substrates. This behavior has enabled their employment not only against microbiota by the means of biofilms, with the relevance of the biofilm-mediated specific interactions between the microbiota and EOs as biocides has been emphasized in this review, but also against fungi and insects. Some limitations have also been critically reviewed, namely their specificity (EOs activity against Gram-positive bacteria is higher than that of Gram-negative bacteria), photochemical stability, pH, and temperature sensitivity.

Nevertheless, modern approaches have already emerged. The use of combined techniques (chemical, physical, biological) and/or active compounds, employment of mixtures of synthetic and natural biocides working in synergy, multitasking solutions (inhibition and removal of mixed species biofilm), and the incorporation of EOs in nanostructured support materials are only a few of the novel directions in research.

A better understanding of the phenomena involved in the essential processes of biofilm formation (e.g., occurrence, growth, and maturation) will make it easier to identify new solutions and design appropriate materials. On the other hand, the constant concern over the side effects of the EOs (toxicity, leakage, odor release, overdosage) may lead to materials with improved characteristics and a lower content in EOs, without limiting their biocide activity. In this context, standardized methods to evaluate disinfection efficiency are highly required.

Nanotechnology employment in EO applications is highly relevant since it allows for the efficient transport of EOs to the sites of action, and their controlled release. Still, the information on this subject is scarce and in-depth studies are necessary. For example, the residual effect generated by nano-encapsulated EOs after the extinction of bacteria, and the effects of the remaining nanocarriers from cosmetic products or implantable devices, need to be further investigated.

However, EOs and their applications represent a very active field of research, with promising prospects. The ongoing research in this field will provide information that will enable scientists and engineers to expand the list of EOs and their range of applications. A close collaboration between research groups and industrial facilities will accelerate technology transfer, thereby reducing the time from the laboratory design stage to end-user application.

## Figures and Tables

**Table 1:** EOs with antibiofilm activity.

EOs	Plant Species	Bacteria	Antibiofilm Activity	Ref.
Clove oil, tea tree oil, cinnamon oil	*Syzygium aromaticum*, *Melaleuca alternifolia*, *Cinnamomum zeylanicum*	SA, PA	IBF	[142]
Thymol, carvacrol, eugenol	-	ST	BE	[143]
Oregano oil, *T. zygis* oil, rosemary oil	*Origanum majorana*, *Thymus zygis*, *Rosmarinus officinalis*	EC	IBF	[144]
Cinnamon oil, eugenol	*Cinnamomum zeylanicum*	SA, PA	IBF, BE	[145]
*Achillea biebersteinii* oil	*Achillea biebersteinii*	SA, EC, PA, SE	IBF	[146]
*Mentha pulegium* oil	*Mentha pulegium*	AB	IBF	[147]
*Cymbopogon citratus* oil	*Cymbopogon citratus*	SM, CA	IBF	[28]
*Calamintha nepeta*, *Foeniculum vulgare*, *Ridolfia segetum* oils	*Calamintha nepeta*, *Foeniculum vulgare*, *Ridolfia segetum*	PA	IBF	[11]
Lemongrass oil	*Cymbopogon flexuosus*	SA, CA	BE, IVBGE	[148]
*Origanum majorana*, *Rosmarinus officinalis*, *Thymus zygis* oils	*Origanum majorana*, *Rosmarinus officinalis*, *Thymus zygis*	SA	IBF, BE	[149]
*Plectranthus barbatus* oil	*Plectranthus barbatus*	PA, CV	IBF, VI, IVBGE	[150]
*Cupressus sempervirens* oil	*Cupressus sempervirens*	KP	IBF, BE	[151]
Thyme, oregano, and cinnamon oils	-	*Acinetobacter*, *Sphingomonas*, *Stenotrophomonas*	IBF	[46]
Clove oil	-	*Aspergillus niger*	IBF	[152]
Eucalyptus oil	*Eucalyptus globolus*	SA	IBF, BE	[153]
*Lippia sidoides* oil	*Lippia sidoides*	SA	BE	[154]
Citrus oil	*Citrus reticulata*	PA	IBF	[155]
*Pogostemon heyneanus*, *Cinnamomum tamala* oils	*Pogostemon heyneanus*, *Cinnamomum tamala*	SA	IVBGE	[156]
*Thymbra spicata oil*	*Thymbra spicata*	PA	IBF, BE	[157]
Spearmint oil	-	*Hafnia alvei*	IVBGE	[158]
*Thymus vulgaris* oil	*Thymus vulgaris*	PF	IVBGE	[159]
*Pimpinella anisum*, *Cinnamomum zeylanicum*, *Syzygium aromaticum*, *Cuminum cyminum* oils	*Pimpinella anisum*, *Cinnamomum zeylanicum*, *Syzygium aromaticum*, *Cuminum cyminum*	SA, SE, EF, EC, PA, CA, KP, *Streptococcus pyogenes*, *Aeromonas hydrophila*, *Proteus mirabilis*	IBF, BE	[160]
Carvacrol, eugenol	-	*Pectobacterium*	IVBGE	[161]

Abbreviations: *Staphylococcus aureus* (SA), *Escherichia coli* (EC), *Pseudomonas aeruginosa* (PA), *Chromobacterium violaceum* (CV), *Staphylococcus epidermis* (SE), *Acinetobacter baumannii* (AB), *Streptococcus mutans* (SM), *Candida albicans* (CA), *Candida dubliniensis* (CD), *Pseudomonas putida* (PP), *Listeria monocytogenes* (LM), *Enterococcus faecalis* (EF), *Salmonella typhimurium* (ST), *Klebsiella pneumonia* (KP), *Pseudomonas fluorescens* (PF); BE = biofilm eradication, IBF = inhibition of biofilm formation, IVBGE = inhibition of virulence factor- and biofilm-related gene expression, RVF = reduction in virulence factors, VI = violacein inhibition.

**Table 2:** Biocide activity of some EOs in wood protection and preservation.

Vegetal Source of Essential Oils	Biocide Activity	Reference
Cinnamon, cassia	active against termites; fungicide (similar to wood tar oil)	[162]
*Origanum vulgare* (oregano); *Acorus calamus* (sweet flag); *Syzygium aromaticum* (cloves- flower buds); *Satureja hortensis* (summer savory); *Betula* tree species (birch)	fungicide: active against fungal organisms such as *Coniophora puteana*, *Trametes versicolor* mildews: *Aspergillus niger*, *Penicillium brevicompactum* (tests on wood specimens from beech tree species)	[165]
*Anethum graveolens* (dill weed); *Salvia rosmarinus* (rosemary); *Cymbopogon* species (lemongrass); *Geranium* species (geranium), *Melaleuca alternifolia* (tea tree)	efficient up to 100% as termites’ repellent (testing activity against *Reticulitermes flavipes*—Kollar)	[163]
Coniferous species belonging to the family *Cupressaceae* (namely, juniper, and cypress); tea tree (*Melaleuca alternifolia*); *Eucalyptus* species from the myrtle family *Myrtaceae*; softwood species *Cupressus nootkatensis* (yellow cedar)	fungicide, insecticide	[170,171,172,173]
*Cinnamomum cassia* (cinnamon); *Geranium* species, *Lavandula* species (lavender), *Origanum vulgare* (oregano); *Thymus vulgaris* (thyme)	fungicide	[174]
Trees and shrubs from *Meliaceae* family (mahogany family), e.g., *Melia azedarach* (white cedar)	activity against viruses and insects	[175]
Cloves—aromatic flower buds of the tree *Syzygium aromaticum*; thyme—*Thymus vulgaris*; mint—*Mentha* species; lemongrass—*Cymbopogon* species; cinnamon—*Cinnamomum cassia*; rosemary—*Salvia rosmarinus*; oregano—*Origanum vulgare*	activity against viruses and insects	[176]
Vetiver—a perennial bunchgrass (*Chrysopogon zizanioides*) patchouli—a bushy perennial herb (*Pogostemoncablin*) orange (*Citrus sinensis*)—from which oil is extracted as a by-product of orange juice production by centrifugation	active against termites (*Coptotermes formosanus*)	[177,178,179]
*Trachyspermum ammi* (ajowan or thymol seeds); *Anethum graveolens* (dill weed); *Geranium* species (geranium); *Cymbopogon* species (lemongrass); *Salvia rosmarinus* (rosemary); *Melaleuca alternifolia* (tea tree); *Thymus vulgaris* (thyme)	active against mildews: *Aspergillus niger*, *Penicillium chrysogenum*, *Trichoderma viride* active against decay fungi: *Gloeophyllum traheum*, *Postia placenta*, *Trametes versicolor*	[180,181]
*Asteraceae* shrub (*Artemisia monosperma*); coniferous tree Mediterranean cypress (*Cupressus supervirens*); lemon tree (*Citrus limon*); coniferous tree from cypress family (*Thuja occidentalis*); Peruvian pepper tree (*Schinus molle*); rose geranium shrub (*Pelargonia graveolens*)	efficient fungicide activity against fungi-degrading wood	[182]
Formosan juniper (*Juniperus formosana*)—extracts from leaves are highly efficient antifungal agents; fruits are also an effective source of compounds with fungal activity	against decay fungi	[183]
*Cinnamomum osmophloeum* Kaneh	100% antifungal efficiency (*Coriolus versicolor* and *Laetiporus sulphureus*)	[184]
Flax seeds—*Cinnamomum cassia* (cinnamon bark); *Citrus sinensis* (citrus peels); tung tree *Vernicia fordii* (tung seeds); *Aleurites moluccana* (nut of kukui tree); non-edible olive oil (called lampante olive oil; here extracted from Istrian white olives variety)	bactericide, fungicide, and insecticide (termites, nematodes)	[185,186,187,188,189]

**Table 3:** Essential oils in food packaging applications.

EO/Main Active Compound/Plant	Formulation	Application	Observations	Ref.
Cinnamon (cinnamaldehyde)	sodium alginate film	ham	Cinnamon migrates through membrane faster than winter-mint.	[214]
*Cinnamonum cassia* (Chinese cinnamon)	solution	fruits and vegetables preservation	Active against *Stenotrophonomonas maltophilia* and *Bacillus subtilis*, and three Penicillium spp., *S. maltophilia*, and *B. subtilis* on bread, carrots, potatoes, sweet potatoes, and apples (testing in situ).	[215]
Cinnaon, clove (cinnamaldehyde, eugenol)	tapioca starch-PHA film	bread	High amount of clove EO is required. Cinnamon film is bactericide; fit for baking.	[216]
Clove (eugenol)	gelatin-chitosan film	fish	Bactericide effects.	[217]
Oregano (carvacrol)	edible films in whey protein isolate formulation	wrapping for Queso Blanco cheese	The edible films strongly limited the oxidation of lipids and hampered the growth of yeasts and mildews.	[218]
Lemon (citral), lemon verbena	chitosan coating	fish (rainbow trout) packaging	Active against *Psychrotrophic* bacteria and *Enterobacteriaceae.*	[219]
Bergamot, oregano	hydroxypropyl methylcellulose films	edible coatings for fresh ‘Formosa’ plum	Effective for storage in low concentrations (2–5% EOs).	[220]
Cinnamon, mustard	zein-based coatings	preservation of cherry tomatoes	Populations of *S. typhimurium* decreased; decrease in the tensile strength of coatings; no ascorbic acid formed during storage.	[221]
Oregano	polypropylene (PP) and polyethylene terephthalate (PET) film	antimicrobial (*E. coli*) packaging films for cheese	Packaging film with oregano EO in different concentrations (0, 4, 6 and 8%) were active against two *Escherichia coli* O157:H7 strains.	[222]
Lemon	chitosan coating	strawberry—control fruit fungal decay	Chitosan–lemon oil coatings had a negative impact on fruit’s aroma profile.	[223]
Basil (eugenol)	PVA/chitosan active films	food packaging	Homogenous transparent films with strong interaction between components, UV-vis and water resistant, and radical scavenging against DPPH.	[209]
Black cumin	PET film assembled with antimicrobial chitosan and alginate coatings incorporating EO	antimicrobial packaging film for chicken meat	Active against *Staphylococcus aureus* and *Escherichia coli.*	[224]
Mint	chitosan film	food packaging	Biodegradable film incorporated EO and showed improved properties (e.g., tensile strength, opacity, water vapor barrier). Active against *Staphylococcus aureus*, *Salmonella typhimurium*, *Klebsiella pneumoniae*, *Bacillus subtilis*, and *Pseudomonas aeruginosa*.	[225]
Clove	chitosan nanoparticles	fungicide; controlled release to extend the shelf life of the fruits and vegetables in fresh state.	Highly active against *Aspergillus niger*.	[152]
Orange	chitosan/fish gelatin composite films	active preservative packaging	EO presence yielded in films with low mechanical properties (tensile strength, elastic modulus), reduced water solubility, moisture content, and water vapor permeability, but with increased elongation at break and hydrophobicity. Moreover, EO enhanced the inhibitory activity of coatings, being efficient when testing free radicals (DPPH and ABTS) and different pathogens (*Staphylococcus aureus* and *Escherichia coli*).	[211]
Pomegranate, *Thymus kotschyanus* (pulegone)	chitosan–starch composite film	packaging that may extend the shelf life of beef	Antibacterial properties against several spoilage bacteria and *Listeria monocytogenes.*	[226]
Grass (*Cymbopogon flexuosus*)	EO microemulsion	prevent the adherence of bacterial species to stainless steel and PP surfaces	Active and efficient when testing microorganisms such as *L. monocytogenes*, *S. aureus*, and *S. typhimurium.*	[227]
Clove (*Syzygium aromaticum*; eugenol), cinnamon (*Cinnamomum zeylanicum*, cinnamaldehyde)	solution	biocide to *S. aureus* strains separated during milking process applied to cows presenting mastitis	Significantly limited the formation of the biofilm on various surfaces (polystyrene, stainless steel).	[141]
Litsea (*Litsea cubeba*, citral)	polyvinyl acetate (PVA) film	antimicrobial packaging material	Citral coatings have showed effectiveness as bactericide agents when testing *Escherichia coli* and *Staphylococcus aureus* and may be employed in hurdle technology applications.	[228]
Clove	composite films based on PLA/graphene oxide nanosheets and EO	food safety and preservation packaging	Graphene oxide increased the thermo-mechanical and barrier properties of the composite films, while EO improved their flexibility and bactericide effects (*Staphylococcus aureus*, *Escherichia coli*).	[229]
Black pepper, ginger	PVA/gum Arabic/chitosan composite films incorporating EO	wound bandages and preservation packaging for food	The films were considerably flexible and thermostable, resistant to break. The incorporation of EOs made films active and efficient when testing *Bacillus cereus*, *Staphylococcus aureus*, *Escherichia coli*, and *Salmonella typhimurium*.	[230]

**Table 4:** Some applications of EOs in restoration and preservation of cultural heritage.

Application	EOs/Plant	Observations	Reference
Cement grit, ceramic, marble	EO mainly composed of phenolic monoterpene carvacrol, extracted from *Thymus capitata* (Malta)	Effective against green algae and cyanobacteria.	[271]
Natural cultural heritage sites: caves	EOs of two *Origanum vulgare* plants (wild and R)	Active against fungi (phylum *Ascomycota*): *Sordariomycetes* (11 isolates), *Eurotiomycetes* (7 isolates), and *Dothideomycetes* (1 isolate).	[272]
Limestone and granite blocks from Royal Tombs in Tanis, Egypt	Various EOs (fennel, lemon, marjoram, rosemary, and spearmint)	Active against yeast colonies (*Candida albicans*, *C. lipolytica*, *Lodderomyces elongsporus*, and *Saccharomyces cerevisiae*).	[273]
Mosaic tesserae (“Casa di Leda”, Greco–Roman site, Solunto, Italy)	EOs from *Thymus vulgaris* and *Origanum vulgare*	Effective against bacteria (*Bacillus*), fungi (*Alternaria*, *Aspergillus*), cyanobacteria (*Chroococcus*), and green algae (*Chlorella*).	[274]
Outdoors stone from heritage sites in South Korea (Royal Tombs of the Joseon Dynasty) and Laos (Vat Phou temple)	Eugenol isolated from volatile clove extracts	Great antifungal activity.	[275]
Wooden artworks	EOs from *Origanum vulgare* or *Thymus vulgaris*	Fungal colonization (*Aspergillus flavus*) or insect infestation (*Anobium punctatum*).	[251]
religious artifacts made of wood and stone	EOs from cinnamon bark and leaves of the genus *Cinnamon*	Phototrophs (*Chlorella* sp., *Chroococcus* sp.); microfungus (*Torula* sp.); brown rot fungus (*Coniophora puteana*).	[276]
Marble statue restauration (statue of Silvanus, National Archeological Museum of Florence)	EOs (carvacrol, eugenol, cinnamaldehyde and thymol) of *Coridothymus capitatus* L., *Syzigium aromaticum* (L.) Merr. and L.M. Perry, *Cinnamomum zeylanicum* Blume, and *Origanum vulgare* subsp. Hirtum Link Ietsw	Tested on cyanobacteria, fungi, and lichens.	[277]
Tholu Bommalu typical Indian leather puppets (International Puppets Museum “Antonio Pasqualino”, Palermo, Italy)	EOs with complex composition extracted from *Thymus vulgaris* and *Crithmum maritimum*	Excellent inhibitory activity against isolated bacteria (*Bacillus*, *Georgenia*, *Streptomyces*, *Ornithinibacillus*).	[278]
Cleaning and restauration of artworks	Free EOs, namely *Lavandula luisieri* and *Calamintha nepeta*, encapsulated by co-precipitation with β-cyclodextrin	High activity against *Aspergillus niger*, *Cladosporium* spp., *Penicillium* spp., *Rhodotorula* sp. and *Arthrobacter* sp. strains, and *Aspergillus* spp., *Epicoccum nigrum*, *Fusarium* spp.	[279]
Preservation of waterlogged archeological wood	EOs from cinnamon bark, wild thyme, and common thyme	Effective against cellulolytic fungal strains: *Chaetomium* sp., *Fusarium* sp., *Aspergillus japonicus*, and *Stachybotrys chartarum*.	[280]
Documents on different supports	EOs of the plants *Pimpinella anisum* L. (anise), *Syzygium aromaticum* L. (clove), *Cuminum cyminum* L. (cumin), *Allium sativum* L. (garlic), *Laurus nobilis* L. (laurel), *Citrus sinensis* (L.) Osbeck (orange sweet), and *Origanum vulgare* L. (oregano)	Clove, garlic, and oregano oils showed the highest antibacterial activity against *Enterobacter agglomerans* and *Streptomyces* sp. Clove and oregano oils were effective against *Bacillus* sp. EOs of sweet orange and laurel had a negative activity against fungi, while anise and clove were highly effective against fungi. Isolated bacterial strains: *Bacillus polymyxa*, *Bacillus cereus*, *Bacillus thuringiensis*, *Bacillus* sp., *Enterobacter agglomerans*, and *Streptomyces* sp. The fungal strains: *Aspergillus niger*, *Aspergillus clavatus*, *Penicillium* sp., and *Fusarium* sp.	[281]
Documentary heritage (photographic papers, glass slides, books, and a map made on paper)	Two essential oils of *Origanum vulgare* L. and *Thymus vulgaris* L.	Antifungal activity against *Scopulariopsis* sp. and *Fusarium* sp. isolated from documentary heritage.	[282]
Historical art craft materials (fibers, woods, dyes, stone)	EO of the wild *Thymus capitatus* (L.) Hofmgg. and Lk. (northern Sicily)	High antimicrobial activity of the oil detected against *Bacillus subtilis*, *Fusarium oxysporum,* and *Aspergillus niger.*	[283]
Artworks	EOs from tea tree, *Calamintha nepeta*, and *Allium sativum* L. extracts	Effective against *Bacillus subtilis*, *Micrococcus luteus*, *Penicillium chrysogenum,* and *Aspergillus* spp.	[250]
Mural paintings (15th–16th century fresco wall paintings from granite churches located in northern Portugal)	EOs from *Rosmarinus officinalis*, *Foeniculum vulgare*, *Citrus lemmon*, *Ocimum basilicum*, and *Salvia officinalis*	Isolated fungal strains: *Alternaria alternata*, *Alternaria tenuissima*, TZ_10.2.2_ (possibly *Pestaloptia* sp.) and TZ_8.2.2_ (possibly *Penicillium* sp.). Basil EO was the only one able to inhibit the growth of all fungal isolates.	[284]
